# Extracting key information from historical data to quantify the transmission dynamics of smallpox

**DOI:** 10.1186/1742-4682-5-20

**Published:** 2008-08-20

**Authors:** Hiroshi Nishiura, Stefan O Brockmann, Martin Eichner

**Affiliations:** 1 Theoretical Epidemiology, University of Utrecht, Yalelaan 7, 3584CL, Utrecht, The Netherlands; 2 Department of Epidemiology and Health Reporting, Baden-Württemberg State Health Office, Nordbahnhofstr. 135, D-70191 Stuttgart, Germany; 3 Department of Medical Biometry, University of Tübingen, Westbahnhofstr. 55, D-72070 Tübingen, Germany; 4 Department of Infectious Disease Epidemiology, Robert Koch Institute, Seestr. 10, D-13353 Berlin, Germany

## Abstract

**Background:**

Quantification of the transmission dynamics of smallpox is crucial for optimizing intervention strategies in the event of a bioterrorist attack. This article reviews basic methods and findings in mathematical and statistical studies of smallpox which estimate key transmission parameters from historical data.

**Main findings:**

First, critically important aspects in extracting key information from historical data are briefly summarized. We mention different sources of heterogeneity and potential pitfalls in utilizing historical records. Second, we discuss how smallpox spreads in the absence of interventions and how the optimal timing of quarantine and isolation measures can be determined. Case studies demonstrate the following. (1) The upper confidence limit of the 99th percentile of the incubation period is 22.2 days, suggesting that quarantine should last 23 days. (2) The highest frequency (61.8%) of secondary transmissions occurs 3–5 days after onset of fever so that infected individuals should be isolated before the appearance of rash. (3) The U-shaped age-specific case fatality implies a vulnerability of infants and elderly among non-immune individuals. Estimates of the transmission potential are subsequently reviewed, followed by an assessment of vaccination effects and of the expected effectiveness of interventions.

**Conclusion:**

Current debates on bio-terrorism preparedness indicate that public health decision making must account for the complex interplay and balance between vaccination strategies and other public health measures (e.g. case isolation and contact tracing) taking into account the frequency of adverse events to vaccination. In this review, we summarize what has already been clarified and point out needs to analyze previous smallpox outbreaks systematically.

## Background

Smallpox epidemiology has the longest and richest history in investigating the mechanisms of spread and in evaluating the effectiveness of vaccination [1,2]. Modern epidemiological methods have developed in parallel with smallpox control practice, and consequently, the disease had already been eradicated before statistical and epidemiological techniques for analyzing infectious disease outbreaks had sufficiently matured.

Although the world is free from smallpox, researchers continue revisiting smallpox epidemiology and virology with recent techniques. In the aftermath of the 9-11-2001 attack, the awareness of the threat of bioterrorism has grown significantly [3]. Mathematical models and computer simulations have been developed to design and optimize public health measures against re-introduced variola virus, the causative agent of smallpox [4-17]. These models are based on different epidemiological assumptions of smallpox. For example, assumptions about the number of secondary transmissions before onset of illness had not been not carefully validated in earlier mathematical modelling studies [16,17]. Accordingly, the policy implications of these models differed widely, and thus the necessity arose to capture the basic mechanisms of smallpox transmission precisely [6,18]. To date, it has been demonstrated that transmission dynamics and intervention strategies cannot be modelled without sufficiently quantifying the detailed intrinsic mechanisms by using observed data [6,19,20]. Because of the global eradication, we have had to maximize the use of historical data to estimate nearly all biological and epidemiological parameters that are needed to optimize interventions [21].

This review article has two purposes. The first purpose is to summarize the issues that have been clarified in recent mathematical and statistical studies and to discuss the relevant policy implications. The second purpose is to specify what important aspects of smallpox epidemiology remain unknown and to suggest how these could be addressed by analyzing historical records. In the following section, we first give a technical overview of the use of historical data and then present some examples of quantification. Subsequently, we summarize the basic concept and interpretation of the transmission potential and the resulting implications for vaccination strategies. The paper concludes with a summary of the findings, emphasizing the importance of systematically analyzing historical datasets.

## Review

### Extracting key information from historical data

Although historical data have frequently been revisited using modern statistical techniques to identify epidemiological determinants of smallpox, many key issues remain unknown in spite of great efforts. To clarify important aspects of smallpox epidemiology, it remains necessary to maximize the use of historical data. To understand their usefulness and to avoid common pitfalls, we briefly discuss technical issues in utilizing historical publications.

### Issues to consider when looking at historical smallpox data

In the following, we list key points to be remembered whenever we statistically extract information from the historical literature. As we may not be able to find all the answers to the following questions in a single historical data set, we may have to combine different data sets or to merge in information from laboratory experiments.

#### (A) Were all cases caused by variola virus?

As cases could not be confirmed virologically before the middle of the 20th century, it is crucial to know on what observations historical diagnoses were based. It was not uncommon to misdiagnose chickenpox cases as smallpox [22,23]. In the older literature, it sometimes even remains unclear which kind of "plague" was being described [24,25]. Ascertainment of diagnostic methods is one of the biggest challenges in utilizing historical outbreak data.

#### (B) Clinical documentations and time-varying medical trends

Similarly, clinical classifications of smallpox have been revised over time [1,26-28]. The definition of severe smallpox has varied greatly even in the 20th century. Vaccines have continuously been improved [29], and we still do not even know from where the vaccinia virus emerged and when it started to be used as a smallpox vaccine [30]. It is necessary to identify and to select the most useful sources of literature, in order to understand which classification in a given publication was adopted and which type of vaccine was most likely to have been used in the population described.

#### (C) Pathogenicity and virulence of the variola virus

Classically, smallpox was classified into two different types according to the observed case fatality. The traditional form of smallpox, referred to as variola major, was believed to have a case fatality of 20% or more. A milder form of the disease, variola minor, with a case fatality of 1% or less was first reported in the late 19th century in South Africa, then it was observed in European countries and finally in Brazil [1,31-33]. Variola minor accounted for the majority of cases in the early 20th century in the United States, where it remained the only form of smallpox from the 1930s until its eradication [34]. The epidemiology of variola minor and its interplay with variola major have only partly been clarified [35,36]. There are clear genetic differences between variola major and minor, supporting the taxonomic distinction; recently, the virulence of variola virus has also been attributed to detailed genomic information [37,38]. However, if case fatality was a major criterion in determining the virulence of variola virus, the outcome of these laboratory studies may have been distorted by the vaccination history of cases and maybe also by other factors. Epidemiological clarification of this point still remains an open question.

#### (D) Definition of the reported events

When extracting information on the incubation and infectious periods (or similar parameters describing the epidemiological characteristics), it is crucial to know how the time of infection (which cannot be observed directly) and the onset of disease were defined. There were two traditional ways to define the onset of smallpox: onset of fever or appearance of rash. If the period from onset to recovery is documented, it is important furthermore to identify what "recovery" stands for (e.g. recovery from pyrexia or solidification/disappearance of rash).

### Extracting data from historical publications

The foregoing list does not cover all common pitfalls. Tackling historical data requires not only statistical techniques, but also understanding of the social history and the background of the cases. Moreover, as noted above, we often have to draw conclusions with implications for public health decision-making using combined data from different sources. Identifying the most useful and important data and addressing key questions are major parts of an essential process to shed light on the mechanisms of transmission and spread of smallpox. In the next two sections, we review studies on parameter estimation that use historical records and predominantly originate from our previous studies. The following case studies were conducted, carefully accounting for common technical problems as listed above when looking at the historical data.

### Intrinsic transmission process of smallpox

To understand the spread of smallpox, it is essential to know the intrinsic transmission process, i.e. after what time symptoms appear, when secondary transmission occurs, and how severe the disease will be. Although basic, such knowledge of the intrinsic transmission process already allows us to assess whether public health interventions in the event of a bioterrorist attack can contain smallpox by means of mass vaccination or by a combination of contact tracing, quarantine and isolation [6,19]. As practical examples, here we briefly discuss basic methodologies and recent findings concerning the incubation period, the infectious period and the case fatality.

### Incubation period of smallpox

The incubation period is defined as the time from infection to onset of disease [39]. Usually, symptoms of smallpox appear 10–14 days after infection [40]. The knowledge of the incubation period distribution enables us to determine the appropriate length of quarantine [41]. 'Quarantine' here refers to physical separation of healthy individuals who were exposed to cases. In the practice of outbreak investigations, the time of exposure is sometimes determined by contact tracing. Historically, the suggested length of smallpox quarantine tended to be 14–16 days, based on professional experience and an accumulation of epidemiological data, but not on an explicit statistical analysis of the incubation period distribution.

Restricting the movement of exposed individuals for longer than the maximum incubation period ensures the effectiveness of quarantine measures. Unfortunately, the length of the incubation period requires knowing the exact time of infection, and thus can only be determined for cases who were exposed for a very short period of time. In addition, the maximum observed incubation period clearly depends on the sample size: the larger the sample size, the more likely we are to find cases whose incubation periods exceed the previously known maximum. The number of smallpox cases with well-known incubation period (e.g. documented patients who had been exposed for a single day only) is limited in historical records. The problem of stating a maximum incubation period can be circumvented by fitting a statistical distribution to the data. This distribution allows a time point to be determined beyond which the onset of further cases becomes extremely unlikely (e.g. the time after infection until which 99% of the patients develop symptoms). If the incubation period follows a lognormal distribution with mean, *μ*, and standard deviation, *σ *(of the variable's logarithm), the probability density of observing an incubation period of length *t*_i _is given by

(1) $$f(t_{i};\mu ,\sigma^{2})=\frac{1}{t_{i}\sigma \sqrt{2\pi }}\exp\left(-\frac{-{\left(\ln(t_{i})-\mu \right)}^{2}}{\sigma^{2}}\right)$$

We can estimate the parameters *μ *and *σ *from a dataset of *n *known incubation times *t*_i _by maximizing the likelihood function

(2) $$L(\mu ,\sigma^{2})=\prod_{i=1}^{n}f(t_{i};\mu ,\sigma^{2})$$

**Figure 1:**
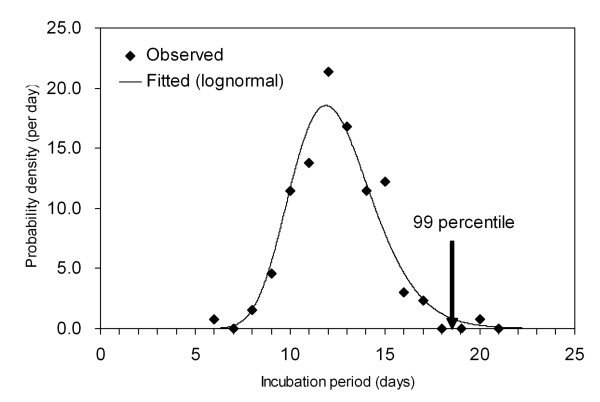
**Incubation period distribution of smallpox fitted to a lognormal distribution (n = 131)**. The vertical arrow indicates the maximum likelihood estimate of the 99th percentile of the incubation period [42]. The median and the coefficient of variation are 12.5 days and 18.0%, respectively.

Figure 1 shows the frequency distribution of the incubation period of smallpox, which was estimated from 131 cases of smallpox who were exposed only for a single day [42]. The mean incubation period is 12.5 days (SD 2.2 days). The 99th percentile is 18.6 days with a 95% confidence interval (CI) ranging from 16.8 to 22.2 days. This indicates that a quarantine of 23 days ensures that more than 99% of infected individuals will develop symptoms before being released.

### Infectious period of smallpox

The infectious period has traditionally been defined as the period in which pathogens are discharged [43]. It presently refers to the period in which infected individuals are capable of generating secondary cases. Knowledge of the infectious period allows us to determine for how long known cases need to be isolated and what should be the latest time point after exposure at which newly infected individuals should be in isolation.

One approach to addressing this issue is to quantify how the pathogen load changes over time using the most sensitive microbiological techniques (e.g. polymerase chain reaction), but such observations are usually limited to the period after onset of symptoms. Several attempts have been made to measure the distribution of the virus-positive period of smallpox cases [44,45], but sample sizes were small and only very few samples could be obtained during the early stage of illness. Moreover, linking "virus-positive" results to the probability of causing secondary transmission is difficult without further information (e.g. frequency, mode and degree of contact).

Another way of addressing this complicated issue is to determine the frequency of secondary transmission relative to disease-age, i.e. the time since onset of fever [46]. An estimate of the relative infectiousness is obtained by analyzing historical data in which it is known who acquired infection from whom. The known transmission network permits serial intervals to be extracted, i.e. the times from symptom onset in a primary case to symptom onset in a secondary case [47-49]. Given the length of the serial interval *s *and the corresponding length of the incubation period *f*, the disease-age *l *from onset of symptoms to secondary transmission satisfies

(3) *s *= *l *+ *f*

Considering the statistical distributions for each length results in a convolution equation:

(4) $$s(t)=\int_{0}^{t}l(t-\tau )f(\tau )d\tau$$

The frequency *l*(*t*-*τ*) of secondary transmission relative to disease-age can be back-calculated by extracting the serial interval distribution *s*(*t*) from a known transmission network, and by using the incubation period distribution *f*(*τ*) given above. If we have information on the length *t*_i _of the serial interval for *n *cases, the likelihood function is given by

(5) $$L=\prod_{i=1}^{n}s(t_{i})=\prod_{i=1}^{n}\int_{0}^{t_{i}}l(t_{i}-\tau )f(\tau )d\tau$$

The parameters that describe the frequency of secondary transmission relative to disease-age can be estimated by maximizing this function. A similar method has been applied to estimate the number of HIV-infections from AIDS incidence [50].

**Figure 2:**
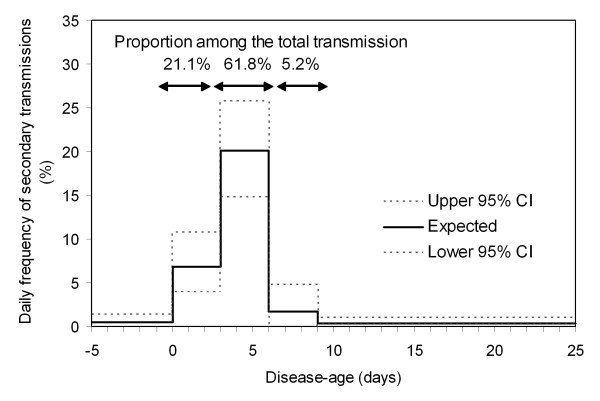
**Relative frequency of secondary transmissions of smallpox by disease-age**. Expected daily frequency of secondary transmissions with corresponding 95% confidence intervals [46]. The percentages indicate the fraction of transmissions among all transmissions that occurred in the given intervals. The disease-age *t* = 0 denotes the onset of fever.

Figure 2 shows the back-calculated infectiousness of smallpox relative to disease-age [46]. In the following text, day 0 represents the onset of fever. Before onset of fever (i.e. between day -5 and day -1) altogether only 2.7% of all transmissions occurred. Between day 0 and day 2 (i.e. in the prodromal period before the onset of rash) a total of 21.1% of all transmissions occurred. The daily frequency of passing on the infection was highest between day 3 and day 5, yielding a total of 61.8% of all transmissions. These estimates help determine the latest time by which cases should be in isolation. If each primary case infects on average six individuals, and if the effectiveness of isolation is 100%, the isolation of a primary case before the onset of rash reduces the expected number of victims to 6 × (0.027 + 0.211) = 1.428. Optimal isolation could, therefore, substantially reduce the number of secondary cases, and the outbreak could quickly be brought under control by additional countermeasures (e.g. contact tracing [6,51]).

### Case fatality

Case fatality is the proportion of deaths among those developing the disease. It is particularly important to understand the case fatality of smallpox in order to estimate the magnitude of the disaster in the event of a bioterrorist attack. It may also be of practical importance to predict the burden of hospital admissions and burials in such an event. The case fatality of smallpox was systematically reviewed during the Eradication Programme [52], showing extremely high crude estimates of 26% and 36% in East-Pakistan and Madras, respectively, but suggesting a wide geographical heterogeneity. Recent studies attributed part of this heterogeneity to viral genomic differences [37,38], but many of the previously published mathematical models simply assumed an overall estimate of 30% for unvaccinated cases.

**Figure 3:**
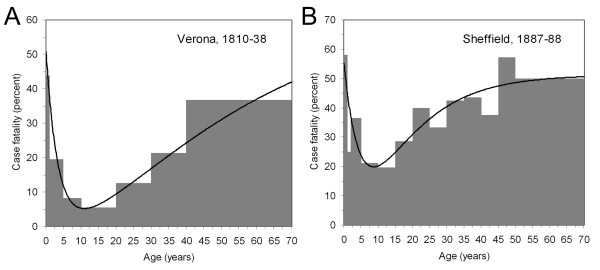
**Age-specific case fatality of smallpox**. Observed (grey bars) and fitted (continuous line) age-specific case fatalities of unvaccinated cases in (A) Verona, Italy, 1810–1838 [53,55] and (B) Sheffield, UK, 1887–8 [54].

Various factors influence case fatality, most importantly previous vaccination history (which will be discussed in the Section on public health interventions) and the age at infection. Following a previous study by Dietz and Heesterbeek [53], we assume the following parametric model for the age-specific case fatality of smallpox:

(6) *c*(*a*) = *α *exp(-*βa*) + *γ*(1 - exp(-*δa*))^2^

where *α*, *β*, *γ *and *δ *are parameters that need to be estimated. If we have a dataset with *M*_i _deaths and *N*_i _survivors of age *a*_i_, the likelihood function is

(7) $$L(\alpha ,\beta ,\gamma ,\delta )=\prod_{i}c{\left(a_{i}\right)}^{M_{i}}{\left(1-c(a_{i})\right)}^{N_{i}}$$

where *a*_i _is a mid-point of age group *i*. Figure 3 shows age-specific case fatality estimates of unvaccinated cases in Verona, Italy, from 1810–38 and Sheffield, UK, from 1887–88, respectively [54,55]. The age-specific case fatality of smallpox can be depicted as a U-shaped curve that peaks in infancy and in old age. Smallpox case fatality also depends on other biological factors of the host such as pregnancy [56], which increases the case fatality from 12.7% (estimate for non-pregnant healthy adults; 95% CI: 11.2–14.3) to 34.3% (95% CI: 31.4–37.1) [57]. Underlying diseases (e.g. cancer, diabetes mellitus, HIV infection and medical immunosuppression for transplantation) could further increase the case fatality.

Above, we have presented the three most important components of the intrinsic transmission process. Each of them plays a key role in determining the optimal intervention strategy. We showed some basic applications of utilizing likelihood functions [58], but various other statistical approaches have been taken which were motivated by similar epidemiological interests. These include the applications of non-linear models [59] and of Bayesian techniques [60].

### Transmission potential

In addition to the epidemiological parameters that characterize the natural history of smallpox, we have to know the most important summary measure of transmission, the basic reproduction number, *R*_0_, in order to design and optimize public health interventions. *R*_0 _is defined as the average number of secondary cases arising from a single index case in a fully susceptible population in the absence of interventions [61,62]. Here, we discuss the concept of *R*_0 _and its estimation, starting with its historical development. Then we use the basic reproduction number to assess the eradication threshold of smallpox by means of mass vaccination.

### R_0 _and vaccination

Smallpox is the disease with the longest history in theoretical modelling. During the 18th century, the famous mathematician Daniel Bernoulli modelled the spread of smallpox and assessed the effectiveness of the variolation practice (variolation was the precursor of vaccination, consisting of the inoculation of variola virus) [53,63]. Moreover, the earliest formulation and calculation of *R*_0 _may have been stimulated by smallpox [64]. The earliest concept of *R*_0 _and the relevant insights into the effectiveness of smallpox vaccination are revisited in the following.

**Figure 4:**
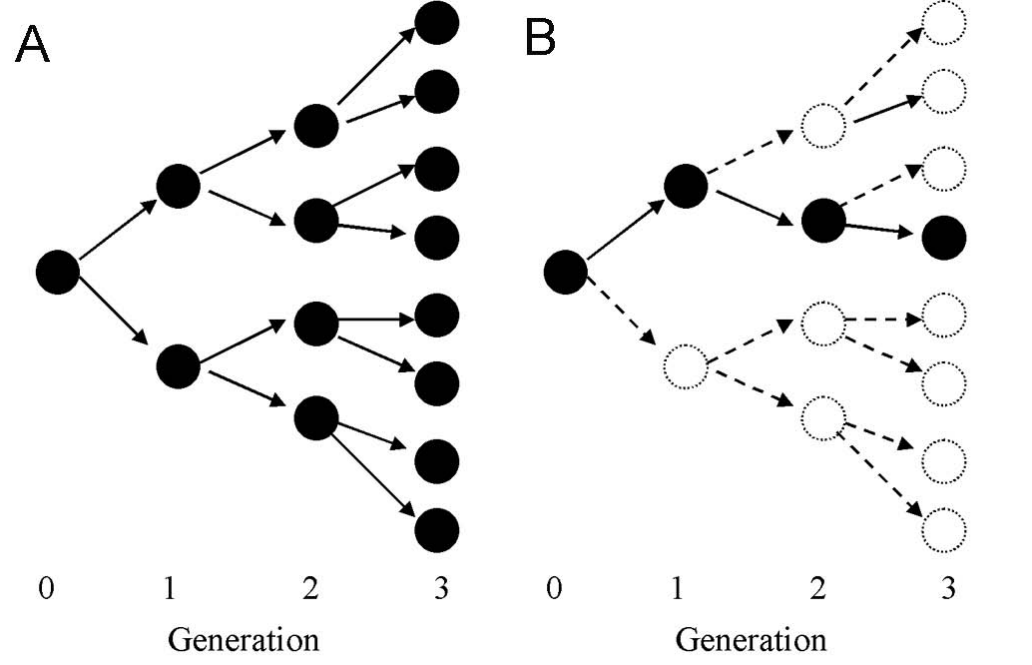
**Theoretical initial courses of smallpox outbreaks following a geometric growth**. A. The infection tree (i.e. transmission network) of smallpox is shown by generation, following equation (8). For simplicity, *R*_0 _is assumed to be 2. B. Infection tree under vaccination. Vaccination is assumed to reduce the number of secondary transmission by 50%, and thus only 1 case in each generation is expected.

Figure 4a shows the result of the simple mathematical model developed by Theophil Lotz in the late 19th century [64]. If a single primary case generates on average *R*_0 _= 2 secondary cases, and if we ignore for the sake of simplicity the depletion of susceptible individuals, the number of cases grows geometrically. If there are *a *index cases in generation 0, the expected numbers of cases in generations 1, 2, 3, ..., *n *will be

(8) $$aR_{0},aR_{0}^{2},...,aR_{0}^{n},$$

respectively. Following Lotz's example of *R*_0 _= 2, and assuming a single index case (*a *= 1), we expect 2, 4, 8, ..., 2^n ^cases in the subsequent generations. Although the model ignores variations in the number of secondary transmissions (which are deemed particularly important for directly transmitted diseases [65]), the process described reasonably captures the essential dynamics of transmission during the early stages of an epidemic.

We now move on to describe various attempts to estimate *R*_0_, summarized in Table 1 together with the underlying key assumptions. Whereas an analysis of long-term temporal dynamics using a mathematical model suggested widely varying estimates of *R*_0_, ranging from 3.5 to 6.0 [66], stochastic models assuming a homogeneous pattern of spread, but ignoring the pre-existing immunity level in the afflicted population, grossly underestimated *R*_0 _as slightly above unity [67]. A revised estimate by a model that accounts for the detailed intrinsic dynamics of smallpox in an initially partially immune population suggested that *R*_0 _is in the order of 6.9 (95% CI: 4.5, 10.1) [18]. This roughly corresponds to an *R*_0 _for which 80–90% of vaccination coverage would allow sufficient herd immunity to be achieved [68,69] (i.e., population-based protection of unvaccinated individuals due to the presence of vaccinated individuals), similarly to Bernoulli's early model, which yielded an estimate of the force of infection that can be translated to *R*_0 _= 6.7 [53].

**Table 1:** Reported estimates of *R*_0 _for smallpox

Location	*R*_0_	Range (min, max)	Assumptions
Unspecified [68,69]	5.0	-	Calculated from proposed goal of vaccination coverage
Abakaliki, Nigeria, 1967 [67]	1.1	(1.0, 1.2)^‡^	Population mixes randomly, initially fully susceptible
Various outbreaks in Europe and the US, 18–20th centuries [66]	3.5–6.0	(3.4, 10.8)	Population mixes randomly, initially fully susceptible
Paris, 17th century [53]	6.7	-	Population is fully susceptible at birth
Abakaliki, Nigeria, 1967 [18]	6.9	(4.5, 10.1)^‡^	Initially partially immune, heterogeneous mixing

^‡^The ranges for the outbreak in Abakaliki are 95% confidence intervals.

*R*_0 _plays a key role in determining the critical vaccination coverage in a randomly mixing population [70]. If *v *= 80% of individuals are protected by vaccination, the average number of secondary cases is reduced to 20%. Following the model of Lotz, the number of cases in each generation is

(9) $$a(1-v)R_{0},a{\left(1-v\right)}^{2}R_{0}^{2},...,a{\left(1-v\right)}^{n}R_{0}^{n}$$

Figure 4b shows the growth of cases when *v *= 50% are protected by vaccination: only a single case is expected in each generation (for *R*_0 _= 2). The number of cases decreases from one generation to the next if (1-*v*)*R*_0 _is less than 1 (cf. equation (9)). In line with this, we can formulate the most fundamental condition of immunization to achieve a sufficiently high herd immunity level. In order to eradicate an infectious disease by vaccination, the fraction protected by vaccination must satisfy [71]

(10) $$v>1-\frac{1}{R_{0}}$$

If *R*_0 _is 6 for smallpox, more than 1-1/6 = 83.3% of susceptible individuals need to be successfully immunized to prevent an epidemic by vaccination alone. Although the pattern of smallpox spread is most probably non-random, equation (10) can be used as an approximation to guide policymaking. If all individuals are vaccinated, *v *can be interpreted as the direct effectiveness of vaccination [72,73]. The effectiveness of smallpox vaccination remained controversial during the early 20th century, partly because of a lack of reliable estimation methods [2], but the methodologies have greatly improved since then [74-78]. During the late 19th century, when vaccine quality was not always ensured, the crude overall effectiveness of vaccination seems to have been higher than 85% [1,21].

### Heterogeneity and behavioural change in relation to historical data

To predict the course and size of an epidemic appropriately, it is critically important to clarify the heterogeneity of transmission. The above-mentioned critical coverage for eradication assumes a randomly mixing population, but it has been established that smallpox spreads for example more easily within households than in the community [79-82]. A theoretical approach to modelling household and community transmission separately has been described [14,83], and a tool that allows the two different levels of transmission to be estimated has been developed [84], but it is very difficult to obtain the necessary estimates from the limited information given in historical records (e.g. detailed household transmission data are always distorted by vaccination). Age-related heterogeneity is yet another important determinant of smallpox epidemiology [85], and spatial patterns of transmission can also influence the success of interventions [86]. Unfortunately, historical records, especially those recorded during the Intensified Smallpox Eradication Programme, are considerably biased (e.g. by individual vaccination histories), and thus it is difficult to address age-related and spatial heterogeneities.

Behavioural changes during an outbreak also have to be clarified to model a bioterrorist attack realistically. It has been suggested that the frequency of contact decreases after the information on an ongoing epidemic is widely disseminated [87-89]. A mathematical model that attempted to incorporate such a declining contact frequency during an epidemic suggested that even gradual and moderate behavioural changes could drastically slow the epidemic [90]. Methods incorporating such changes remain yet to be developed to help public health policy making. A generalized method could perhaps incorporate results of a psychological response survey [91].

### Public health interventions

Given the basic parameters that describe the intrinsic transmission process, we are now able to examine the effectiveness of interventions. In addition to the critical level of mass vaccination that was discussed in the previous section, here we discuss further issues on vaccination strategies and other public health interventions in bioterrorism preparedness.

### Duration of vaccine-induced immunity and partial protection

The degree of protection of vaccinated individuals in the present population is yet another important public health issue. Immunological studies showed that a fraction of previously vaccinated individuals still reacts to exposure with variola virus [92,93], but it is difficult to attribute each kind of immunological response to actual protection against the disease and its severity. Thus, the degree of protection of individuals who were vaccinated 30 to 50 years ago has remained an open question.

As we have previously shown, an epidemiological model that partly addressed the effect of booster events estimated that primary vaccination protected for a median duration of 11.7 to 28.4 years against the disease [94], indicating that most vaccinated individuals in the present community may no longer be protected from contracting smallpox. However, similar estimates also indicate that vaccinated individuals are still protected against severe manifestations and death from smallpox [95]. An analysis of a statistical record of an outbreak in Liverpool from 1902–3 revealed a median duration of protection against smallpox death of 49.2 years (95% CI: 42.0–57.3) [95,96]. This finding (of long-lasting partial protection from severe manifestations) was further supported by statistical analyses of similar historical datasets [94] and of individual case records extracted from historical outbreaks in Australia where booster events were extremely rare [97]. In the event of a bioterrorist attack in the early 21st century, residual immunity could significantly decrease the individual burden of disease. However, the persistence of partial protection does not necessarily imply a positive impact on the population level. Masked symptoms may cause difficulties in case recognition and clinical diagnosis. Although it might be virologically plausible that previously vaccinated cases are less infectious (e.g. due to low levels of virus in their nasopharynx), reduced severity may also permit movements of infectious individuals, worsening the prospects of public health control. The ripple benefit of residual immunity has yet to be clarified.

To understand the complex interplay of all partial effects of vaccination, various biological and social effects must be considered. In theory, vaccination does not only diminish the susceptibility of vaccinated individuals, but also reduces the degree and duration of infectiousness upon infection. The vaccine-induced reduction of infectiousness can be estimated using the household secondary attack rate (SAR), expressed as the ratio of the number of infected household contacts to the number of exposed household contacts [98]. Suppose that SAR_ij _denotes the household secondary attack rate where *i *and *j*, respectively, give the previous vaccination histories of the secondary and primary case (i.e. *i *or *j *= 1 represents previously vaccinated, whereas *i *or *j *= 0 represents unvaccinated individuals). Let us consider the following household transmission data, which were observed in India [79]:

The household SAR caused by unvaccinated cases among unvaccinated and vaccinated contacts were estimated to be SAR_00 _= 40/650 = 0.0615 and SAR_10 _= 11/583 = 0.0189, respectively. Those caused by vaccinated cases among unvaccinated and vaccinated household contacts were SAR_01 _= 10/499 = 0.0200 and SAR_11 _= 2/421 = 0.0048, respectively.

The crude efficacy of vaccine in reducing susceptibility VE_S_, infectiousness VE_I_, and a combined effect of both VE_T _is then estimated by

(11) $$\begin{array}{c} {\text{VE}}_{S}=1-\frac{{\text{SAR}}_{10}}{{\text{SAR}}_{00}}=0.693\\ {\text{VE}}_{I}=1-\frac{{\text{SAR}}_{01}}{{\text{SAR}}_{00}}=0.674\\ {\text{VE}}_{T}=1-\frac{{\text{SAR}}_{11}}{{\text{SAR}}_{00}}=0.923 \end{array}$$

If we make the simplifying assumption that the biological effect of vaccination was identical for all vaccinated individuals, vaccination reduced susceptibility by 69.3%, infectiousness by 67.4%, and the combined effect was 92.3%. Although an effect of vaccination on the duration of disease has rarely been observed and reported, historical epidemiological studies in Dalian, China, suggested that the mean symptomatic period was reduced by 13.7–48.5% if the case was previously vaccinated [21,99].

### Vaccination strategies

Given that the intrinsic dynamics as well as the effects of vaccination are sufficiently quantified, vaccination strategies against smallpox can be optimized. Three issues, of which the epidemiology has been discussed though the quantitative effect has not yet been fully clarified, are discussed in the following: revaccination, ring vaccination, and post-exposure vaccination.

After it became clear in the late 19th century that vaccine efficacy was not perfect and that vaccine-induced immunity waned over time, revaccination was put into practice. Revaccinated individuals were said to have contracted smallpox less often and had much milder manifestations, so that scheduled revaccinations became accepted in the early to mid 20th century [26], but the intervals from primary vaccination to revaccinations and the number of revaccinations varied widely within and between countries, making analytic evaluations very difficult. Accordingly, it is extremely difficult to quantify the effectiveness of revaccination in reducing the chance of smallpox even with statistical techniques in the present day. Crude estimates of the increased protection against smallpox death were obtained for several outbreaks; e.g. for Madras during the 1960s [27], where 87.1% fewer cases died in the revaccinated group than in the group who had only received the primary vaccination (770/3266 vs. 4/132 deaths, respectively), but this revaccination effect only measures what happened to people who were infected in spite of vaccination. (What makes an explicit interpretation of these findings even more difficult was the fact that vaccination in India was made using the rotary lancet, which left a scar even in the absence of "take".)

Vaccination can be combined with the practice of case finding: Ring vaccination is a surveillance containment measure that consists of vaccinating and monitoring all susceptible individuals in a prescribed area around one or several index cases [100]. This combined strategy is deemed more effective than mass vaccination [101], but combinations of vaccination and public health measures have not yet been explicitly evaluated. Ring vaccination was introduced and evaluated mainly in West and Central Africa and in Asia where it was always combined with case isolation [102]. Although it is difficult to exclude the impact of other interventions and to estimate the net effectiveness of ring vaccination explicitly (e.g. the impact of previous vaccinations can usually not be separated [103]), accumulated experience during the Intensified Eradication Programme strongly suggests that ring vaccination (accompanied by vigorous isolation) worked well [101]. The strategy is deemed logically effective in containing localized outbreaks, but it is important to ensure effective contact tracing if we are to rely on ring vaccination alone [9,104].

Vaccination may still be protective if a person has already been exposed to the virus, a procedure referred to as post-exposure vaccination [105,106]. Despite numerous discussions [107], the protective effect of post-exposure vaccination has remained unclear. A historical study from the early 20th century suggests that vaccination within 7 days after exposure is effective [28]. Smallpox textbooks in the 1960s and '70s claimed that 'vaccination within 72 hours almost promises protection' [26,27], a statement roughly consistent with a more recent statistical estimate based on historical data and on several assumptions concerning the hypothetical frequencies of vaccinated and protected individuals [108], and with a laboratory study demonstrating a cell-mediated response within 4 days after exposure [109]. A similar estimate was obtained in a Delphi analysis, which concluded that post-exposure vaccination can be assumed to be 80–93% effective during the first 3 days after exposure and 2–25% thereafter [110]. However, as we have shown, a statistical exercise demonstrates that historical data, which only record cases who developed smallpox after post-exposure vaccination, hardly provide sufficient insight into the effectiveness of post-exposure vaccination [111]. Information regarding the denominator is insufficient for the majority of records (i.e. we do not know how many exposed people who were vaccinated were protected from the disease). Only the effectiveness of vaccination against severe disease upon infection can be estimated from such data: the shorter the interval between exposure and vaccination, the lower the probability of developing severe smallpox. To the best of our knowledge, only one outbreak in Leicester, UK, from 1903–04 provided insight into the protection against disease by post-exposure vaccination [112]: counting from the eruption of the index case, it was reported that none of 210 individuals (0%) who were vaccinated on the first day after exposure, 2 among 359 (0.5%) who were vaccinated on the second day, 5 among 102 (4.9%) who were vaccinated on the third day, and 10 among 116 (8.6%) who were vaccinated on the fourth day or later developed the disease. Although this seems to indicate some degree of protection, the actual efficacy of post-exposure vaccination can only be determined by comparing these findings to observations in a group of individuals who were exposed for exactly the same periods of time, but refused or were denied post-exposure vaccination.

Despite effective vaccination, pros and cons of vaccination practice always need to account for adverse events of vaccination [113]. Vaccine-strain dependent differences in the frequency of adverse events have been reported, and the risk of death due to vaccination has been analyzed in detail only recently [114,115]. Theoretical frameworks reported to date agree with each other that we should not implement pre-attack mass vaccination in order to minimize the number of adverse events. Policy suggestions of mathematical models for post-attack vaccination strategies depend on the specific attack scenarios and need to be investigated further.

### Case isolation and contact tracing

Rather than relying completely on vaccination, recent modelling studies have suggested that an outbreak could be contained by a combination of case isolation and contact tracing [6,14], owing mainly to the characteristics of the intrinsic dynamics of smallpox (e.g. the relatively long generation time and the clear symptoms of smallpox). The importance of monitoring and controlling "contacts" has been highlighted in a historical observation [112] and was also stressed during the Eradication Programme [51,116,117]. A public health system's capability in conducting contact tracing may determine whether or not a smallpox outbreak can be controlled without vaccination. This should also take into account response logistics and the limited number of public health practitioners [104]. A mathematical exercise suggested that the optimal intervention also depends on the initial attack size: whereas an outbreak caused by few cases could easily be controlled by isolation and contact tracing alone, regional (targeted) mass vaccination is recommended if the initial attack size is big and *R*_0 _is large [118].

## Conclusion

This article has reviewed quantifications of the transmission and spread of smallpox using historical data. Although historical data are limited and we cannot answer all questions regarding smallpox epidemiology, many publications are available from previous efforts. However, a systematic listing of surveillance data and/or outbreak reports irrespective of language (e.g. see [57]) still remains a future task. It is essential that historians, smallpox specialists and epidemiologists interact more.

Since the eradication, smallpox deaths have disappeared from the world [119], and hope has arisen that we will succeed in eradicating other infectious diseases. Owing to the conceived threat of bioterrorism, researchers nevertheless have to continue working on smallpox, and we have entered yet another round of discussing the pros and cons of smallpox vaccination. The current debates of preparedness issues are far more complex than mass vaccination, and newer vaccination strategies complicate the balance between individual and community benefits [120]. Once other infectious diseases have been eradicated, we will see similar discussions arise, but before this becomes the case, it is important to make sure that systematically collected data are aggregated and stored for posterity.

## Competing interests

The authors declare that they have no competing interests.

## Authors' contributions

HN reviewed the literature, analyzed the data and drafted an early version of the manuscript. ME reviewed the early version of the manuscript and assisted in editing the manuscript. SOB participated in the writing and revision of the manuscript. All authors have read and approved the final manuscript.
